# Design and Parameter Optimization of Deep Well Rapid Purification System Combining Nanobubble Water Spray and Water Bath/Wire Mesh Carbon

**DOI:** 10.3390/nano16030199

**Published:** 2026-02-02

**Authors:** Xin Zhang, Yixiao Xie, Yong Jin, Xingxin Nie, Zeyu Sun, Lihua Mi, Rui Tao

**Affiliations:** 1 College of Architecture and Energy Engineering, Wenzhou University of Technology, Wenzhou 325000, China; zhangxin17@xauat.edu.cn (X.Z.);; 2 Shaanxi Coal Industry New Energy Technology Co., Ltd., Xi’an 710100, China; 3 School of Resources Engineering, Xi′an University of Architecture and Technology, Xi’an 710055, China

**Keywords:** mining pollution, spray dust suppression, micro-nano bubble water, water bath/wire mesh, performance effect

## Abstract

In order to create a safe and healthy working environment in mines, an issue that urgently needs to be addressed is the rapid discharge of high concentrations of toxic and harmful pollutants after blasting. This paper proposes a deep well rapid purification system based on the combination of nanobubble water spray and water bath/wire mesh carbon, and conducts single-variable optimization tests on the parameters of micro-nano bubble water and the atomizing nozzle. The wet spray fiber grid and carbon adsorption network form in sequence and verify the purification experiment under the clear optimal parameters. The results show that the micro-nano bubble water is used as the spray medium, and a high-pressure nozzle with a diameter of 0.4 mm is also used. The water supply pressure of the nozzle is 3.0 MPa, the wet spray fiber grid uses a double-layer 10-mesh metal wire, and the carbon adsorption network uses 5 mm activated carbon fiber cotton as the optimal parameter for the deep well rapid purification system. Under these conditions, the efficiency of total dust and exhalation dust reduction is 72.90% and 79.17%, respectively, and the purification efficiency of CO, H_2_S, and SO_2_ reaches 84.39%, 78.75%, and 55.54%, respectively. This study provides reference data for efficient pollution reduction in mines and has high practical value.

## 1. Introduction

In recent years, with the continuous increase in demand for mineral resources and the increasing depletion of shallow mineral reserves, deep mining has become a key trend in global mining development [1,2]. However, the specific geological and mining conditions of deep mines make the dust pollution faced by mining operations complex, stubborn, and hazardous [3]. In key processes such as deep well blasting and excavation, a large amount of dust aerosols containing harmful substances such as free silica and heavy metal ions are instantly generated [4,5]. These dust particles not only suspend and diffuse for a long time in the confined space of the mine but also further intensify the retention and spread of dust due to the environmental characteristics of high wind pressure, high humidity, and high temperature in deep mines [6]. Long-term exposure to high dust concentration environments makes workers highly susceptible to occupational diseases such as pneumoconiosis, posing a serious threat to their life and health [7,8]. At the same time, the accumulation of dust can also reduce underground visibility, interfere with the precise operation of mechanical equipment, increase equipment wear and failure probability, and exacerbate the risk of safety accidents [9]. Therefore, investigating how to achieve efficient dust reduction is currently a key research focus.

Ventilation and dust removal technology, as the core method of mine dust control, has been widely studied and applied [10]. Traditional mine ventilation and dust removal technology mainly relies on three modes, forced ventilation, extraction ventilation, and mixed ventilation [11,12,13], which dilute and discharge dust by constructing an airflow field. Among them, forced ventilation can quickly deliver fresh airflow to the working face, but it can easily cause dust to diffuse into the deep part of the tunnel [11]. Extraction ventilation can directly extract dusty airflow from the working face, with relatively high dust removal efficiency, but it has the disadvantages of high wind resistance and high energy consumption [12]. Although hybrid ventilation combines the advantages of both methods, it has strict requirements for the layout design of the ventilation system. In complex tunnel networks in deep wells, problems such as airflow short circuits and multiple air leakage points are prone to occur, making it difficult to achieve precise and efficient dust control [13]. In addition, traditional ventilation techniques often focus on dilution and exhaustion, lacking active dust capture mechanisms. Faced with the purification needs of high concentration and fine-grained dust after deep well blasting, they often exhibit shortcomings such as a slow purification speed, high energy consumption costs, and unstable treatment effects. In addition, as the ventilation distance of mines continues to increase, it also brings about problems such as increased resistance in the ventilation system, more air leakage points, insufficient effective air supply to working points, and increased ventilation costs [14]. In order to increase the effective air volume at the working point, it is possible to establish a new ventilation system; however, this method is too costly and difficult to implement on site. Alternatively, it is possible to increase the power and air volume of the main fan at the mine entrance and exit; however, this method also has drawbacks, such as increased energy consumption and excessive losses during transmission. The actual increase in air volume at the air point is not significant.

Given the limitations of traditional ventilation technology, controllable circulation ventilation technology is the most effective measure for accelerating the discharge of gun smoke from the mining site and optimizing the underground environment [15]. Compared with traditional ventilation renovation techniques, controllable circulation ventilation has the advantages of fast effectiveness and low cost, and effectively alleviates problems such as insufficient ventilation capacity in deep well mining, air leakage in the goaf, and the chaotic layout of local fans. It represents a new approach to achieve cooling and energy-saving goals [15]. However, when using controllable circulation ventilation technology, the introduction of a circulating airflow must not cause the concentration of pollutants in the system to exceed the standard, and the air must be effectively purified before introduction [16]. In response, researchers have begun to explore circulating air purification technologies. These include high-pressure spray dedusting technology [17], the bag dedusting system [18], air–water spray dedusting [19], water bath/wire mesh purification technology [20], ventilation spray collaborative dedusting [21], and other methods. Although some progress has been made, there is still a lack of testing and research on air purification systems, including deep well air purification systems.

In addition, water mist dust suppression technology has gradually become an important supplementary method for mine dust control due to its advantages of having low cost, easy operation, and causing no secondary pollution [22]. The core mechanism of water mist dust suppression is to utilize the collision, interception, and agglomeration of water mist particles and dust particles to increase the weight and settlement of dust particles, thereby achieving efficient dust capture [22]. With the development of nanotechnology, nanobubble water spray dust suppression technology has emerged, becoming a research hotspot in the field of water mist dust suppression [23]. Nanobubble water contains a large number of nanoscale bubbles with a diameter less than 50 μ m, which have characteristics such as a large specific surface area, slow rising speed, high dissolved oxygen content, and strong interfacial activity. When in contact with dust particles, it can not only achieve dust capture through mechanical collision but can also enhance the wetting and agglomeration effect of fine-grained dust through the micro-jet effect generated by bubble rupture. Most of the existing studies have explored the nanobubble water spray dust suppression and water bath/wire mesh carbon dust suppression as independent technologies, focusing on the parameter optimization and effectiveness verification of a single technology. There is no study that combines the two organically or a deep well rapid purification system that combines nanobubble water spray and water bath/wire mesh carbon.

Based on the above practical problems, this paper proposes combining a micro-nano gas bubble water spray system with a water bath filament carbon system to form a combined air purification device, achieve more efficient dust reduction and harmful gas filtration, and carry out relevant factor analysis and research to optimize the performance parameters of the device. The results of this research can provide reference data for the optimization of deep-well air purification systems, and also have important practical significance for the prevention and control of mining blasting dust and toxicity, as well as the occupational safety of operators.

## 2. Methods

### 2.1. Rapid Air Purification Device

**Figure 1:**
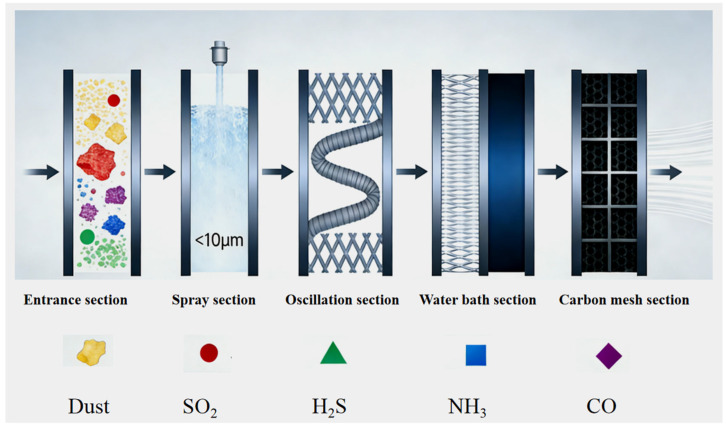
Schematic diagram of purification device.

The rapid air purification device is divided into four sections, combining nanobubble spray dust suppression with water bath/wire mesh carbon purification. The schematic diagram is shown in Figure 1.

Spray section: Micro-nano bubble water is used as the medium. Micro-nano bubble water is rich in extremely fine bubbles with a diameter of less than 10 μm, which are sprayed out through a nozzle and come into contact with dust in the airflow. The bubbles that break during the spraying process decompose into smaller water droplets and adsorb dust.

Oscillation section: A set of fiber fences with different angles are set up on both sides of the tunnel to guide the airflow to flow in an “S” shape inside the tunnel, improve the contact efficiency between the airflow and the purification device, and further strengthen the subsequent purification section’s dust capture and absorption of other gases.

Water bath section: A water spray device is installed on the metal wire mesh, and a downward-flowing water film forms on the metal wire mesh. When the airflow passes through the metal wire mesh, since the H_2_S and SO_2_ contained in the polluted air are easily soluble in water, they can be effectively absorbed by the wet spray fiber grid. Residual mineral dust can be further absorbed in the wet purification system.

Carbon mesh section: A set of activated carbon meshes is installed on both sides of the tunnel to dehydrate and purify the polluted air, adsorbing residual dust particles and CO that does not easily dissolve in water during the airflow.

Finally, under the joint action of the spray dust suppression device and the water bath carbon purification device, the dusty and polluted airflow is filtered and purified.

### 2.2. Experimental Systems

**Figure 2:**
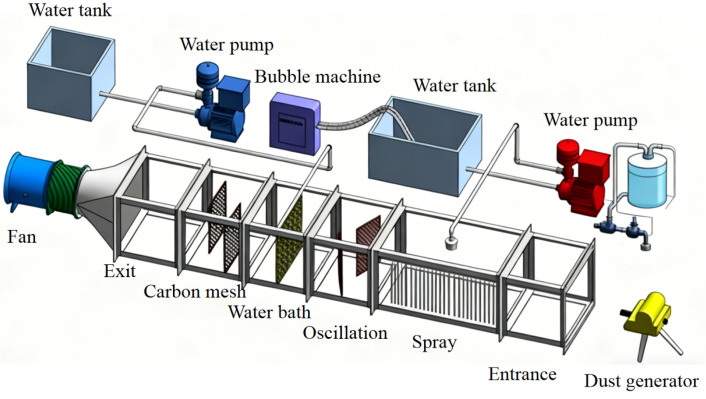
Schematic diagram of rapid air purification system.

The rapid air purification platform consists of a ventilation system, a tunnel model, a dust generator, a micro-nano bubble generation system, a gas atomization system, a water bath carbon filtration system, and a water supply system, as shown in Figure 2. The experiment is conducted at an ambient temperature (25 ± 2 °C) and a relative humidity of 60 ± 5%.

**Figure 3:**
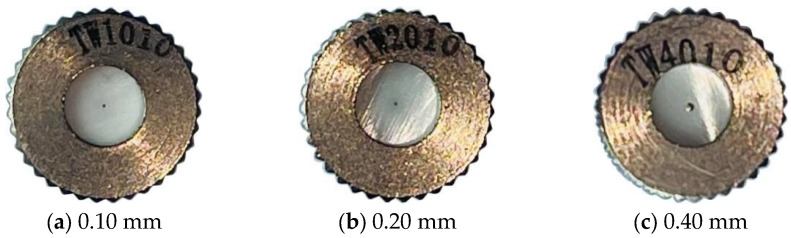
Physical pictures of the nozzle.

The main roadway of the experimental platform is made of a transparent acrylic plate, with a size of 3.5 m × 0.6 m × 0.6 m. The micro-nano bubble spray dust suppression system uses a common three-stage high-pressure nozzle. In the water bath carbon purification system, a 0.6 m × 0.3 m encrypted metal wire mesh is used for the oscillation section, and a 0.6 m × 0.6 m metal wire mesh is used for the water bath section. A high-pressure nylon tube is arranged above the wire mesh, and a uniform outlet is set. The carbon network segment uses a 0.4 m × 0.6 m activated carbon mesh. QJS manganese ore dust from Zhenba, Shaanxi Province, is selected for testing. The nozzle is a three-stage high-pressure nozzle that is commonly used for spray dedusting. The nozzle aperture models are 0.10 mm, 0.20 mm, and 0.40 mm. The structure is shown in Figure 3.

### 2.3. Experimental Apparatus

An REP150 micro-nano bubble generator, supplied by Jiangsu Ruke Environmental Protection Equipment Co., Ltd., Nanjing, China, with a 5 L/min flow rate and 150 W power, is used for preparing the micro-nano bubble water, and a BMSTZWH water pump, with a flow of 7 L/min and a rated working pressure of 6–8 MPa, is used for water supply in the spray system. A variable frequency fan, with an air volume of 1000–4000 m^3^/h, rated power of 0.12–2.2 kW, voltage of 220 V, and total pressure of 59 Pa, is used to maintain stable airflow in the tunnel. Using a direct-reading CCZ1000 dust concentration measuring instrument, supplied by Suzhou Yilian Electromechanical Technology Co., Ltd., Zhangjiagang, China, the measured dust concentration range is 0.1 mg/m^3^–1000 mg/m^3^, with a relative measurement error of ±15%. A portable four-in-one gas detector, capable of detecting the gases CO, SO_2_, and H_2_S with a detection error of ±5% and a response time of 45 s, is also used, in addition to a mechanical electronic anemometer with a wind speed range of 0–8 m^3^/h.

### 2.4. Experimental Protocol

Three groups of tests are performed, including a nanobubble water spray dust suppression test, a water bath/wire mesh carbon purification test, and an optimal combination purification test. The water supply pressure range in the experiment is set to 1.0~3.0 MPa.

**Figure 4:**
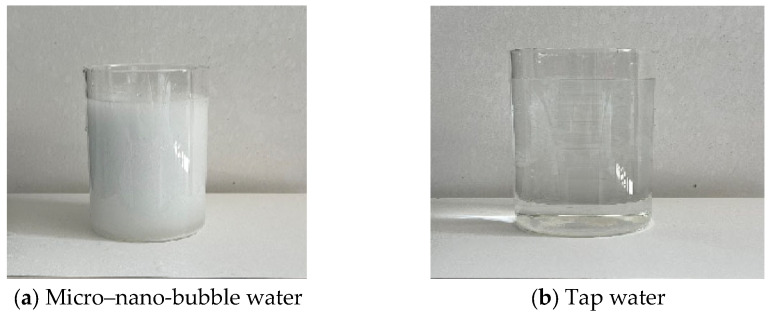
Experimental medium.

The first group of experiments is the nanobubble water spray dust suppression test, and the test materials are shown in Figure 4. The following parameters are used: water type for wet scrubbing: tap water (base) + micro-nano bubble water (spray section); PH of water (spray + water bath sections): 7.2 ± 0.3 (ambient); alkalinity of water: 80 ± 10 mg/L (as CaCO_3_); dissolved oxygen (DO): tap water: 6.5 ± 0.5 mg/L; and micro-nano bubble water: 12.3 ± 0.8 mg/L. By adjusting the water supply pressure, spray medium, and nozzle model, the relationship between the nozzle atomization angle, dust reduction efficiency, and different working conditions is investigated.

The inlet and outlet pipes are all made of high-pressure nylon pipes with an inner diameter of 9.52 mm. The water supply pressure is set to 1.0 MPa, 1.5 MPa, 2.0 MPa, 2.5 MPa, and 3.0 MPa, and the nozzle apertures are 0.10 mm, 0.20 mm, and 0.40 mm. The total dust concentration M_1_ and exhalation concentration m_1_ before dust reduction are measured under different working conditions, and the total dust concentration M_2_ and exhalation concentration m_2_ after dust reduction are also measured. The sampling time is 1 min. The total dust reduction efficiency $\eta_{1}$ and exhalation dust reduction efficiency $\eta_{2}$ are calculated according to Formula (1). To avoid experimental errors, each operating condition is sampled three times continuously, and the average of the three data points is taken for analysis.

(1) $$\eta_{1}=\frac{M_{1}-M_{2}}{M_{1}}\times 100\%$$

where *M_1_* is the total inlet dust sampling mass, g, and *M_2_* is the total outlet dust sampling mass, g.

(2) $$\eta_{2}=\frac{m_{1}-m_{2}}{m_{1}}\times 100\%$$

where *m_1_* is the inlet dust sampling mass, g, and *m_2_* is the outlet dust sampling mass, g.

**Figure 5:**
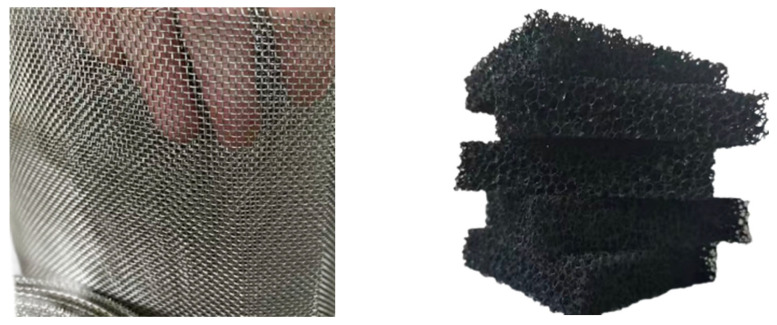
Purification material for water bath silk–charcoal system.

The second experiment is the water bath/wire mesh carbon purification test, which investigates the impact of changing the filter model on the filtration efficiency of cannon smoke. A certain amount of gunpowder is placed in the inlet section for combustion, the wet spray fiber grid model and carbon adsorption network model are adjusted, and the specific parameters are shown in Figure 5 and Table 1. The activated carbon fiber cotton used in the carbon adsorption network is standard unmodified pitch-based activated carbon fiber (ACF) with no impregnation or chemical modification. The following parameters are used: a specific surface area of 1200 ± 50 m^2^/g and a pore structure in which micropores (≤2 nm) account for 75% of total pore volume, mesopores (2–50 nm) for 20%, and macropores (>50 nm) for 5%. To avoid experimental errors, each operating condition is sampled three times continuously, and the average of the three data points is taken for analysis.

The third experiment involves the optimal purification combination. The single optimal operating condition is determined based on the first two sets of experiments to verify the dust reduction efficiency and air filtration efficiency of the rapid air purification device. Similarly, the average of the three datasets is taken for analysis.

## 3. Results and Discussion

### 3.1. Dust Suppression Experiment of Micro-Nano Air Bubble Water Spray

#### 3.1.1. Spray Nozzle Atomization Angle Characteristics

**Figure 6:**
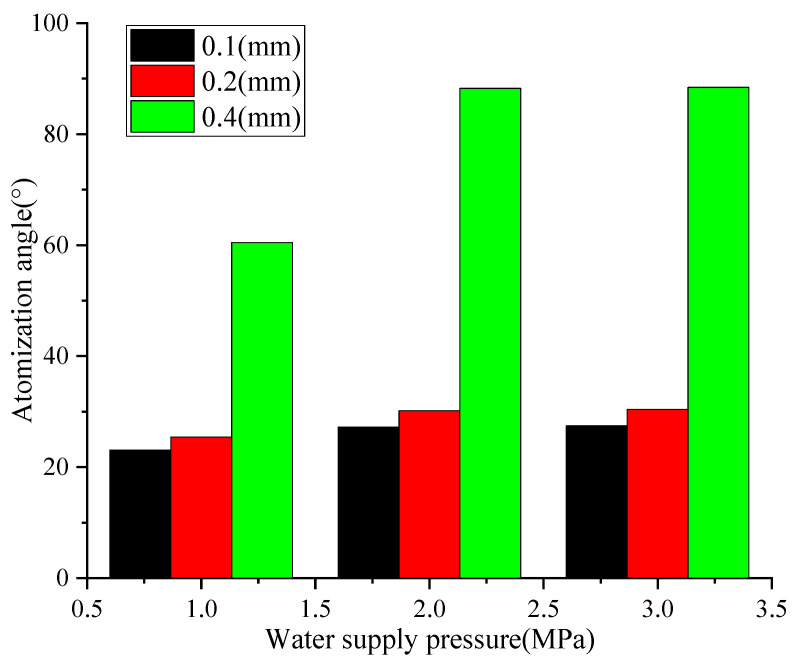
Comparison of different nozzle atomization angles.

The atomization angle variation characteristics of three types of high-pressure nozzles under different water supply pressure conditions are shown in Figure 6.

Figure 6 shows that, as the water supply pressure increases, the atomization angle of these three types of nozzles gradually increases. When the water pressure is within the range of 1.0–3.0 MPa, the 0.4 mm nozzle has the widest coverage, and the atomization angle is always greater than 60°. After the pressure exceeds 2.0 MPa, the increase in atomization angle slows down. When the water supply pressure is 3.0 MPa, the nozzle atomization angle reaches its maximum value of about 90°. This is because, as the pressure increases, the kinetic energy of the fluid ejected from the nozzle increases, and the diffusion range of the droplets formed by overcoming surface tension expands, resulting in an increase in atomization angle [24]. When the pressure reaches 2.0 MPa, the fluid kinetic energy approaches the nozzle structure’s ultimate bearing capacity, and the droplet diffusion boundary effect becomes prominent. The contribution of pressure to the droplet diffusion angle decreases, resulting in a gradual increase in atomization angle. In addition, the larger the aperture, the lower the flow resistance of the fluid inside the nozzle, and the greater the fluid flow rate sprayed per unit time. Therefore, under the same pressure, the atomization angle of a large aperture nozzle is larger. This is consistent with the results in the existing literature [25], verifying the correctness of this paper. The size of the atomization angle directly determines the dust reduction coverage range. A 90° atomization angle can achieve an all-round coverage of the tunnel section, providing optimal conditions for the subsequent dust reduction efficiency of large aperture nozzles.

#### 3.1.2. Influence of Nozzle Aperture on the Dust Suppression Effect of Spray

**Figure 7:**
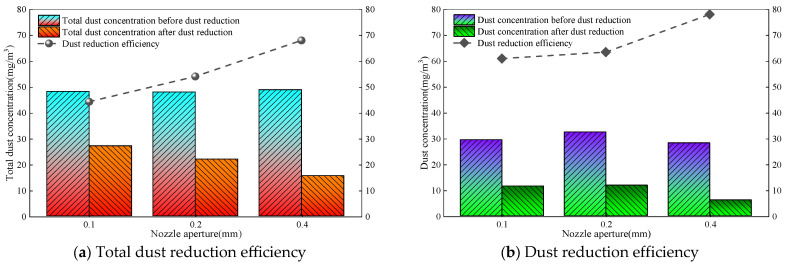
Dust reduction efficiency of different nozzle apertures.

When the wind speed of the roadway is kept at 1.0 m/s, the amount of dust generated is 30 g/min, the spray medium is micro-nano gas bubble water, the water supply pressure is 2.0 MPa, and the nozzle apertures are 0.1 mm, 0.2 mm, and 0.4 mm. The total dust, exhaled dust concentration, and efficiency before and after dust fall are measured as shown in Figure 7.

Figure 7 shows that, when the spray medium and water supply pressure are fixed, the concentration of total dust and respirable dust after dust fall decreases rapidly. With the increase in nozzle aperture, the dust fall efficiency continues to increase. When the nozzle aperture increases, the overall dust reduction efficiency increases from 44.3% to 68%, and the dust concentration reduction efficiency increases from 61% to 78.1%. The dust reduction effect of respiratory dust is more significant. When the nozzle aperture is changed from 0.2 mm to 0.4 mm, the slope of the total dust and inhalation dust reduction efficiency is larger, and the dust reduction efficiency is improved faster. The reason for this result is the synergistic effect of droplet characteristics and the dust capture mechanism [26]. An increase in nozzle aperture results in a significant increase in fluid flow rate and the formation of more droplets under the same water supply pressure, which is positively correlated with the collision probability of dust particles. Especially for fine-grained dust such as dust, the dust collection efficiency improvement is more significant. When the particle size of fog droplets is at the same order of magnitude as that of dust particles, the inertial collision efficiency is the highest, making it difficult for dust particles to flow around the fog droplets, meaning that they are more easily captured [27]. When the aperture is small, the droplet size is too small, and it is prone to flow around when colliding with a large amount of particle dust, which limits the capture efficiency. When the aperture is increased to 0.4 mm, the compatibility between the droplet size and dust particle size is optimized, and the collision efficiency is greatly improved, which is manifested in an increase in the slope of dust reduction efficiency.

#### 3.1.3. Influence of Water Supply Pressure on Dust Suppression Effect of Spray

**Figure 8:**
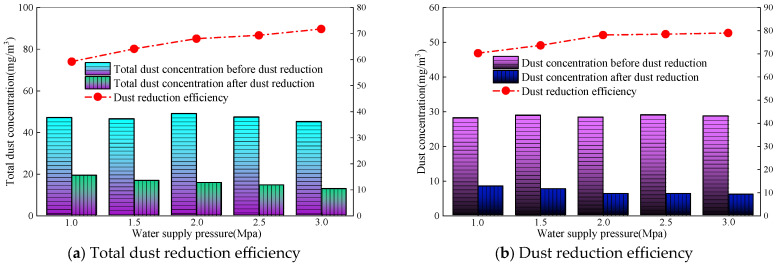
Dust reduction efficiency under different water supply pressures.

Similarly, when the wind speed of the roadway is 1.0 m/s, the amount of dust generated is 30 g/min, the spray medium is micro-nano bubble water, the nozzle aperture is 0.4 mm, and the water supply pressure is adjusted to 1.0–3.0 MPa. The measured concentration and efficiency of total dust and exhaled dust before and after dust fall are shown in Figure 8.

Figure 8 shows that, under the same operating conditions, the dust reduction efficiency of full dust and breathing dust increases as the water supply pressure increases. The total dust concentration drops rapidly after spraying. The total dust concentration before dust fall is about 47 mg/m^3^, and after dust fall is between 12 and 20 mg/m^3^. With an increase in water supply pressure, the total dust fall efficiency continues to rise, from 59.2% to 71.8%. The dust concentration decreases from 29 mg/m^3^ to around 6 mg/m^3^. In the water supply pressure range of 1.0–2.0 MPa, the dust reduction efficiency continues to rise, increasing from 70.23% to 78.1%. However, in the water supply pressure range of 2.0–3.0 MPa, the dust reduction efficiency does not show a significant improvement, and remains stable between 78.1% and 79%. This is because the increase in water supply pressure enhances the shear effect of the fluid inside the nozzle [28], resulting in a decrease in droplet size and a more uniform distribution. The specific surface area of small droplets is larger, and the contact area with dust particles is significantly increased. At the same time, the movement speed of droplets and the collision kinetic energy increases, which can effectively overcome the air film resistance on the surface of dust particles and promote the wetting and bonding between droplets and dust. In addition, the increase in pressure not only increases the number of droplets but also enhances the droplet movement speed, resulting in an increase in both the frequency and intensity of collisions between droplets and dust. For whole dust, the capture of large particle size dust relies on the impact and interception of droplets, and the increase in kinetic energy caused by pressure has a more significant impact. Therefore, the efficiency of whole dust continues to increase; as a fine-grained dust, when the pressure reaches 2.0 MPa, the droplet size of the dust is already small enough (compatible with the dust particle size), and the flow rate can meet the basic capture requirements. However, there is limited room for optimizing the droplet characteristics by increasing the pressure, so the dust efficiency tends to stabilize.

#### 3.1.4. Influence of Spray Medium on Dust Suppression Effect of Spray

**Figure 9:**
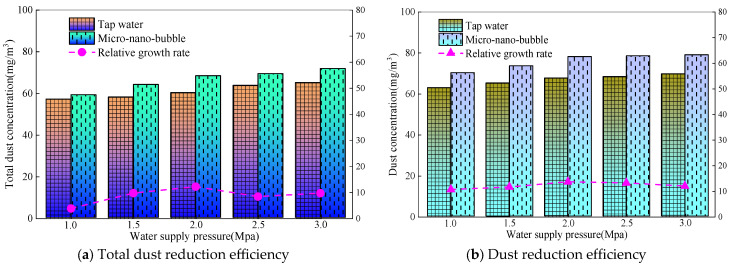
Dust reduction efficiency of different spray media.

Similarly, when the wind speed of the roadway is 1.0 m/s, the amount of dust generated is 30 g/min, the nozzle aperture is 0.4 mm, and the regulated water supply pressure is 1–3 MPa. The spray medium is composed of tap water and micro-nano gas-soaked water, and the concentration and efficiency of total dust and exhaled dust before and after dust fall are shown in Figure 9.

From Figure 9, it can be seen that the total dust and dust removal efficiencies of micro-nano bubble water are significantly higher than that of tap water. At a water supply pressure of 3.0 MPa, the total dust efficiency is 9.4% higher and the dust removal efficiency is 11.8% higher. The main reason for this is that the presence of micro-nano bubbles reduces the surface tension of water [23]. The smaller the surface tension, the easier it is for droplets to spread on the surface of dust particles, and an improvement in wetting effect can significantly enhance the capture efficiency. Compared with the two types of spray media, it is easier to capture dust when the droplet size is close to the dust size. For dust particles floating in the air, a smaller droplet size is more conducive to capture, and when micro-nano bubbles rupture, local micro-jets are generated, and the impact force can break the gas film on the surface of dust particles, promoting the contact between fog droplets and dust [29]. At the same time, the surface of micro-nano bubbles carries negative charges and electrostatically adsorbs with positively charged dust particles, further increasing the probability of capture. Therefore, when micro-nano gas bubbles are immersed in water for spray, the bubbles break down into smaller droplets, which are more likely to capture fine particles such as exhaled dust. Therefore, micro-nano gas bubbles have more obvious dust reduction advantages than exhaled dust, and the efficiency improvement is greater than that of the whole dust.

To sum up, micro-nano bubble water is the spray medium. When the water supply pressure is 3.0 MPa and the nozzle aperture is 0.4 mm, the total dust reduction efficiency of spray is 71.8%, and the dust reduction efficiency of spray is 79%, with the highest efficiency.

### 3.2. Water Bath/Wire Mesh Carbon Air Purification Test

#### 3.2.1. Purification Efficiency of Wet Spray Fiber Grid

**Figure 10:**
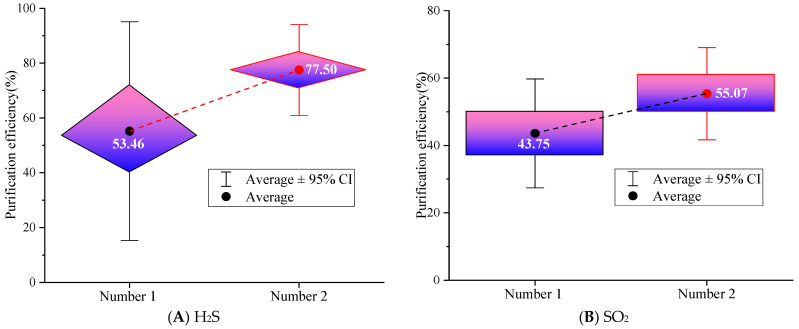
Purification efficiency of different wet spray fiber grids.

Due to the insolubility of CO in water, only the purification efficiency of the wet spray fiber grid for H_2_S and SO_2_ is considered. Figure 10 shows the purification efficiency of the metal wire mesh under different aperture numbers.

From Figure 10, it can be seen that the purification efficiency of the second fiber mesh for H_2_S is 70.83% to 84.17%, which is overall higher than that of the first fiber mesh; meanwhile, the average purification efficiency of SO_2_ by the second type of fiber mesh is 55.07%, which is 11.32% higher than that of the first type of fiber mesh. The main reason is that the purification effect of the wet spray fiber grid depends on the continuous water film formed on the surface of the wire mesh. H_2_S and SO_2_, as water-soluble gases, undergo dissolution reactions through contact with the water film to achieve purification. The aperture of the 10-mesh metal wire is larger, the wire diameter is thicker, and the adhesion area of water flow on the wire mesh is wider, forming a thicker and more continuous water film, avoiding the problem of water film breakage caused by the easy penetration of water flow in the fine 16-mesh wire. The porosity of the 10-mesh wire is higher, the resistance of airflow passing through the wire mesh is smaller, the gas–liquid contact time is longer, and the mass transfer efficiency is higher. At the same time, the water film surface formed by the large-aperture wire mesh is rougher, and the turbulence effect generated by the airflow is enhanced, breaking the gas boundary layer and promoting the diffusion of H_2_S and SO_2_ in the water phase, further improving the absorption efficiency. Overall, the average purification efficiency of the wet spray fiber grid with double-layer 10-mesh metal wire for H_2_S and SO_2_ is 77.05% and 55.07%, respectively. The main reason that the removal of H_2_S exceeds that of SO_2_ is that H_2_S is a weak acid (pKa_1_ = 7.04 [30]), with the dissociation equilibrium H_2_S↔H++HS− at a neutral pH. At pH 7.2 (close to pKa_1_), ~55% of dissolved H_2_S dissociates into ionic species (HS^−^), which are strongly retained in the water film via electrostatic interactions with the metal wire mesh (which are slightly positively charged due to surface oxidation) [31]. This enhances mass transfer and prevents re-emission. SO_2_ is a moderate acid (pKa_1_ = 1.99 [30]) that rapidly dissolves to form H_2_SO_3_ but dissociates primarily into H^+^ and HSO_3_^−^ (pKa_1_ = 1.99) at neutral pH. However, SO_2_’s aqueous solubility (11.3 g/L at 25 °C [32]) is ~30× higher than H_2_S (0.39 g/L at 25 °C [32]), but its removal is limited by reaction kinetics and system constraints (not affinity). Primary references confirm that, in neutral water (pH 6–8), H_2_S removal via wet scrubbing is more efficient than SO_2_, despite SO_2_’s higher solubility, due to H_2_S’s favorable dissociation and ionic retention [31,33].

SO_2_’s dissolution and reaction are kinetically slower than H_2_S. SO_2_ forms H_2_SO_3_, which requires additional time to oxidize to stable sulfate (SO_4_^2−^) via dissolved oxygen [34]. In the original system, DO (6.5 mg/L in tap water) is insufficient to drive rapid oxidation, leading to the reversible desorption of H_2_SO_3_ back to gas-phase SO_2_.

H_2_S, by contrast, undergoes an irreversible reaction in neutral water, i.e., dissolved H_2_S reacts with trace oxygen to form elemental sulfur (S^0^) and water (2H_2_S + O_2_→2S↓ + 2H_2_O) [31]. This reaction removes H_2_S from the aqueous phase permanently, eliminating desorption and enhancing overall efficiency. In the spray section, bubble rupture generates micro-jets that break the gas film on H_2_S aerosol particles, promoting dissolution [29]. Higher DO in micro-nano bubble water accelerates the oxidation of H_2_S to S^0^ [35], while SO_2_’s oxidation to sulfate requires higher DO (>15 mg/L) or chemical oxidants (e.g., H_2_O_2_) not present in the system [34].

#### 3.2.2. Purification Efficiency of Carbon Adsorption Network

**Figure 11:**
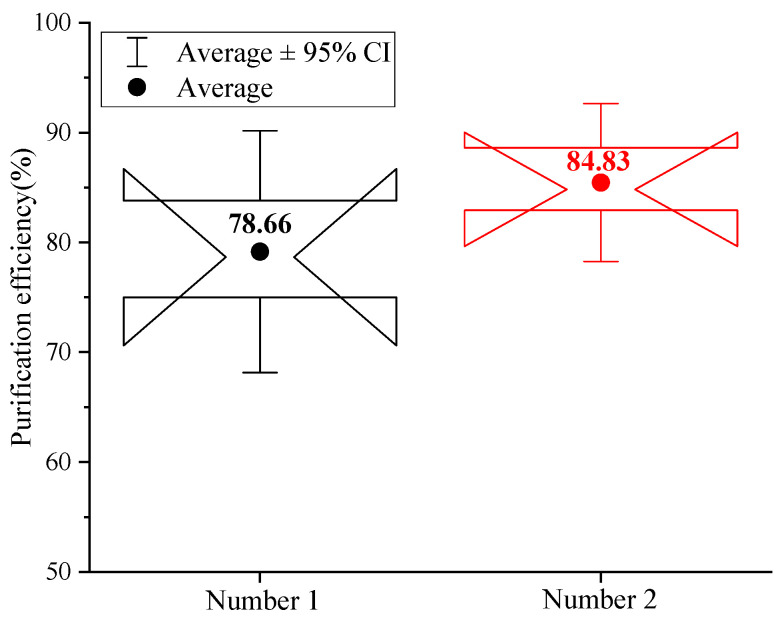
CO purification efficiency of different carbon adsorption networks.

The adsorption capacity of different activated carbon fibers for inorganic gas CO is shown in Figure 11.

According to Figure 11, the average purification efficiency of CO by Carbon Net 2 is 84.83%, which is significantly improved compared with Carbon Net 1. This is mainly because activated carbon fibers have rich microporous and mesoporous structures, and 5 mm thick fiber cotton has a larger specific surface area and pore volume than 3 mm thick fiber cotton. The diameter of CO molecules is about 0.376 nm, which can be captured by the micropores of activated carbon fibers and lead to physical adsorption, and thicker activated carbon fiber cotton provides a longer adsorption path, prolonging the diffusion time of CO gas inside the fiber and increasing the adsorption capacity when reaching equilibrium. Meanwhile, although the surface functional groups of activated carbon fibers have a weak chemical adsorption effect on CO [36], under the dominant role of physical adsorption, a larger contact area and longer diffusion path become key to improving adsorption efficiency. Therefore, a 5 mm thick carbon adsorption network exhibits a better CO purification effect.

In addition, we conduct a blank group test. The same experimental platform is used, but the activated carbon fiber bed is removed to isolate the contribution of other system components (e.g., water bath, spray, and metal wire mesh) to CO removal. Without the activated carbon fiber bed, the CO purification efficiency is consistently <2% (average 1.3 ± 0.4%) across all wind speeds (0.3–1.0 m/s).

This confirms that CO removal is solely attributed to the activated carbon fiber rather than physical absorption by water or interception by other components, eliminating the potential confounding factors in the original study.

### 3.3. Optimal Combination Purification Test

According to the above test results, micro-nano bubble water is selected as the spray medium, a nozzle with a diameter of 0.4 mm is used, and the water supply pressure is 3.0 MPa. The wet spraying fiber grid uses double-layer 10-mesh metal wire, and the carbon adsorption network uses 5 mm activated carbon fiber cotton. The dust purification experiment is conducted under the optimized air purification system configuration, and the results are shown in Table 2.

The results in Table 2 show that, when the wind speed increases from 0.3 m/s to 1.0 m/s, the total dust reduction efficiency increases from 68.57 to 72.90%, and the exhaled dust increases from 73.50% to 79.17%. This is because, as the wind speed increases, the airflow speed in the roadway increases, the diffusion coefficient of dust particles increases, and the collision frequency with spray droplets, water film, and activated carbon fiber increases. Especially for fine-grained dust, it is prone to slow diffusion due to Brownian motion at low wind speeds [37]. The forced convection effect at high wind speeds promotes its contact with the purification medium, resulting in a greater increase in dust efficiency compared with total dust. In addition, due to the “S”-shaped flow channel design of the oscillation section, the residence time of dust in the tunnel is extended, the contact probability between dust and subsequent purification devices is increased, and the mass transfer of H_2_S, SO_2_, and the water film, as well as the adsorption kinetics of CO and activated carbon fibers, is promoted. The carbon network segment adsorbs residual dust and CO through activated carbon, forming a “multi-stage collaborative capture” and enhancing the dust reduction effect. At the same time, the airflow at high wind speeds can promptly carry away captured dust and dissolved gases, increasing the opportunity for dust to come into contact with droplets and mist, and thereby improving dust reduction efficiency. Table 3 shows the purification efficiency of CO, H_2_S, and SO_2_ at different wind speeds.

According to Table 3, when the wind speed is 1 m/s, under the optimal combination, the purification efficiencies of CO, H_2_S, and SO_2_ reach 84.39%, 78.75%, and 55.54%, respectively. The efficiency difference is due to the fact that CO is a non-water-soluble gas that cannot be absorbed by the water film in the water bath section, and its purification relies entirely on the physical adsorption of activated carbon fibers [38]. Notably, 5 mm thick activated carbon fiber cotton provides sufficient adsorption sites, and CO molecules have good size compatibility with activated carbon micropores, resulting in high purification efficiency. H_2_S has strong water solubility; it is partially dissolved in micro-nano gas bubble water in the spray section, and it makes full contact with the water film in the water bath section for dissolution and absorption. At the same time, the physical interception of the fiber grid can also capture some aerosol particles containing H_2_S. Under the dual mechanism, the purification efficiency is only second to CO. Although SO_2_ has strong water solubility, due to its weak molecular polarity, its affinity with water is slightly lower than H_2_S, and the dissolution balance of SO_2_ in water is vulnerable to temperature and pressure [39]. Under the test environment, the retention time of water film in the spray section and water bath section is limited, and some SO_2_ is discharged with the airflow before it is completely dissolved. Therefore, the purification efficiency is relatively low, but more than 55% of the purification effect is still achieved through multi-stage cooperation. For the special nature of underground mining operation environment, systematic optimization and improvement should be carried out according to the differentiated needs of different places [40,41,42,43,44], providing strong guarantees for effectively controlling the stability and safety of the mining working environment.

## 4. Conclusions

This paper proposes to combine the micro-nano gas bubble water spray system with the water bath/wire mesh carbon system to form a combined air purification device. The parameters of the micro-nano gas bubble water and the atomization nozzle, and the wet spray fiber grid and the carbon adsorption network, are optimized by a single-variable test in turn, and the purification experiment is verified when the optimal parameters are clear. The following conclusions have been obtained.

(1) The atomization angle of high-pressure nozzles is ranked as aperture 0.4 mm > aperture 0.2 mm > aperture 0.1 mm; when the water supply pressure is 3.0 MPa, the atomization angle of high-pressure nozzles with aperture 0.4 mm can reach a maximum of 90°.
(2) In terms of the dust reduction efficiency of spray, micro-nano bubbles are better than tap water, and micro-nano bubbles can enhance the dust reduction effect of high-pressure nozzles. The larger the nozzle aperture, the greater the liquid flow rate sprayed, and the more significant the dust reduction effect; the higher the water supply pressure, the greater the dust reduction efficiency. At the same water supply pressure and spray medium, when the pore diameter is 0.4 mm and the water supply pressure is 3.0 MPa, the dust reduction efficiency of the nanobubble water is the highest, reaching a maximum of 71.8% for total dust reduction efficiency and a maximum of 79% for exhalation dust reduction efficiency. The micro-nano bubble more clearly enhances the dust reduction in respirable dust.
(3) Wet spray fiber grids with larger apertures are more likely to form large water films between them, expanding the surface area in contact with H_2_S and SO_2_, which is beneficial for the more effective absorption of water-soluble harmful gases in the air. Under the same operating conditions, thicker activated carbon fibers have more pores and larger contact surfaces, resulting in better adsorption efficiency for CO.
(4) The optimal parameter combination for the rapid air purification system is as follows: micro-nano air bubble water is used as the spray medium, and a high-pressure nozzle with a diameter of 0.4 mm is used. The water supply pressure of the nozzle is 3.0 MPa, the wet spray fiber grid uses a double-layer 10-mesh metal wire, and the carbon adsorption network uses 5 mm activated carbon fiber cotton. Under this optimal configuration, the efficiency of total dust and exhalation dust reduction is increased to 72.90% and 79.17%, respectively, and the purification efficiency of CO, H_2_S, and SO_2_ reaches 84.39%, 78.75%, and 55.54%, respectively, providing technical support for the rapid treatment of complex pollutants after deep well blasting.

## 5. Limitations and Future Research Directions

However, this study has certain limitations. First, the experiment was conducted based on a small-scale transparent tunnel model, failing to simulate the complex real deep well conditions (such as high temperature, high humidity, high wind pressure, and intricate tunnel networks), which may lead to deviations between the experimental results and on-site application effects. Furthermore, the characterization of nanobubbles (including the detection of size distribution, potential, and other indicators before and after the nozzle) was not provided. Additionally, the parameter optimization only adopted single-variable tests, ignoring the interaction effects among key parameters, and the tests were limited to specific types of mineral dust (QJS manganese ore dust) without verifying the system’s adaptability to other mineral dusts or more complex pollutant components, resulting in the insufficient universality of the system. There is a lack of long-term operational testing to evaluate the stability of components like nozzles, wire meshes, and activated carbon fibers, as well as the single-variable (OFAT) sequential optimization approach. While this design simplified the identification of individual parameter effects and enabled the preliminary screening of feasible ranges, it ignored potential interactions between key variables (e.g., nozzle aperture vs. water supply pressure, or wire mesh aperture vs. airflow velocity) that are inherent to atomization and wet scrubbing systems. Such unmodeled interactions may lead to the identification of a local optimum rather than a global optimum. Therefore, future research will focus on constructing a simulation platform for real deep well working conditions and conducting on-site industrial tests to enhance the engineering applicability of the technology. Multivariate optimization methods will be adopted to explore the interaction mechanisms between parameters and verify the system’s purification efficiency for diverse pollutants to improve its universality. In addition, a response surface methodology with a central composite design to systematically model parameter interactions will be employed to identify the global optimum and further improve system efficiency. Finally, long-term operational tests will be performed to optimize the system design, and research will be conducted on intelligent system applications (such as real-time monitoring and adaptive adjustment of purification parameters) to provide more comprehensive and reliable data to support the optimization and promotion of deep well air purification systems.

## Figures and Tables

**Table 1:** Water bath silk–charcoal system material.

Type	Size	Number	
		1	2
Wire mesh	0.6 m × 0.6 m	Aperture—16 mesh	Aperture—10 mesh
Activated carbon mesh	0.4 m × 0.6 m	Thickness—0.03 m	Thickness—0.05 m

**Table 2:** Dust purification efficiency under different wind speeds.

Wind Speeds (m/s)	Total Dust Concentration (mg/m^3^)		Efficiency (%)	Dust Concentration (mg/m^3^)		Efficiency (%)
	Before Purification	After Purification		Before Purification	After Purification	
0.3	34.7	10.4	68.57	25.8	5.8	73.50
	38.2	9.1		30.4	7.3	
	41.5	16.8		20.9	6.9	
0.5	32.9	9.9	69.88	28.7	7.5	74.28
	36.4	12.8		19.5	4.7	
	43.8	11		31.2	8.4	
0.7	39.1	10.1	71.10	32.1	7.9	77.24
	45.6	15.7		24	5.4	
	33.3	8.8		29.3	6.2	
1.0	28.3	7.1	72.90	18.8	3.3	79.17
	39.5	9.3		24.2	5	
	45.3	14.8		31.7	7.7	

**Table 3:** Purification efficiency of gas.

Wind Speeds (m/s)	CO Concentration (ppm)		Efficiency (%)	H_2_S Concentration (ppm)		Efficiency (%)	SO_2_ Concentration (ppm)		Efficiency (%)
	Before Purification	After Purification		Before Purification	After Purification		Before Purification	After Purification	
0.3	42.3	8.1	80.17	5.3	1.1	73.59	18.3	8.7	53.48
	55.7	11.4		7.8	2.8		20.7	9.6	
	47.8	9.5		6.2	1.4		19.5	8.9	
0.5	51.4	9.3	82.15	8.5	2.6	75.18	21.2	11.0	53.52
	48.9	6.1		4.7	0.6		17.8	7.9	
	53.1	12.2		9.0	2.8		22.0	9.5	
0.7	40.8	4.4	83.25	6.4	1.6	77.96	19.9	9.7	54.14
	58.3	13.5		8.2	2.2		20.4	8.8	
	46.0	7.5		4.9	0.7		18.6	8.5	
1.0	60.0	11.6	84.39	6.9	1.6	78.75	19.1	7.0	55.54
	40.6	6.0		4.9	1.7		20.9	11.2	
	43.2	5.5		5.1	0.3		20.4	8.8	

## Data Availability

The data presented in this study are available from the corresponding author upon request.
